# Mapping Segregation Patterns of Hospital Care among Patients with Limited English Proficiency

**DOI:** 10.1007/s10903-024-01630-5

**Published:** 2024-09-13

**Authors:** Kathy Sliwinski, April J. Ancheta, K. Jane Muir, Karen B. Lasater

**Affiliations:** 1 Center for Health Outcomes and Policy Research, University of Pennsylvania School of Nursing, Philadelphia, PA USA; 2 National Clinician Scholars Program, Craig-Dalsimer Division of Adolescent Medicine, Leonard Davis Institute of Health Economics, University of Pennsylvania, Children’s Hospital of Philadelphia, Philadelphia, PA USA; 3 Center for Health Outcomes and Policy Research, School of Nursing, National Clinician Scholars Program, University of Pennsylvania, Leonard Davis Institute of Health Economics, University of Pennsylvania, Philadelphia, PA USA; 4 Center for Health Services and Outcomes Research, Northwestern University Feinberg School of Medicine, Chicago, USA

**Keywords:** Limited English proficiency, LEP, Language barriers, Hospital segregation

## Abstract

Individuals with limited English proficiency (LEP) are disproportionately more likely to experience suboptimal care outcomes compared to English-proficient individuals, attributed to multi-level social determinants of health, including the quality of the hospital where LEP patients are more likely to receive care. Evidence demonstrates that racial minority patients are more often admitted to lower-quality hospitals serving high proportions of minority patients, despite living closer to higher-quality hospitals. Less is known about where individuals with LEP reside, where they seek hospital care, and the quality of care in these hospitals. Using Geographic Information Systems (GIS) methods, we developed a density map characterizing residential patterns of the LEP population across zip code tabulation areas in New Jersey and designated hospitals as high, middle, or low-LEP volume. We described differences in 30-day hospital wide readmission rates for hospitals across varying LEP volume status using Centers for Medicare and Medicaid Services Hospital Care Compare Data. Most hospitals in ZCTAs with higher LEP populations serve a high proportion of LEP patients (i.e. their patients’ demographics are reflective of the community in which they are located). However, our results also show instances in which LEP patients may be forgoing receiving care at closer hospitals to instead receive care at further-distanced, high-LEP volume hospitals. significant. High-LEP volume hospitals have higher 30-day hospital wide readmission rates (20.1%) compared to middle (15%) and low (11.3%)-LEP volume hospitals (*p* < .001), indicating lower quality of care within high-LEP volume hospitals.

## Introduction

Hospitalized individuals with limited English proficiency (LEP)––those with limited ability to read, speak, write, or understand English [1]––experience disproportionately worse healthcare outcomes (e.g., more readmissions, longer hospital stays) compared to their English-proficient counterparts [2, 3]. These outcome disparities can be attributed to multi-level social determinants of health, including unaddressed language barriers, poor health insurance coverage, and disparities in the quality of hospitals where LEP patients are more likely to receive care [4, 5]. Existing literature demonstrates patterns of segregation in hospital care delivery, for example, racial minority patients are more often admitted to lower-quality hospitals despite living closer to higher-quality hospitals [6, 7]. This study characterizes patterns of hospital care segregation and hospital quality relative to where individuals with LEP reside.

## Methods

Using ArcGIS Pro software, we developed a density map characterizing residential patterns of the LEP population across zip code tabulation areas (ZCTAs) in New Jersey (NJ) in 2016. The proportion of LEP individuals per ZCTA was provided by 2016 American Community Survey data, which identifies individuals that report speaking English “less than very well.” We determined the proportion of LEP patient index admissions per hospital, provided by the NJ Discharge Data Collection data. We divided the sample of 73 NJ hospitals into sextiles, characterizing sextiles 1–3 as low (0.02–6.4% LEP patient index admissions), 4/5 as middle (6.6–17.4%), and 6 as high-LEP volume hospitals (17.5–88.1%). Hospitals were then plotted as a secondary layer to our LEP population density map to assess congruence between where LEP individuals reside and where they seek hospital care. To assess each hospital’s quality of care, we used the Centers for Medicare and Medicaid Services Hospital Care Compare data, which provided 30-day hospital-wide readmission rates, a risk-standardized all-condition measure for unplanned readmissions within 30 days of discharge. We performed an analysis of variance to examine differences in 30-day hospital-wide readmission rates for hospitals across varying LEP volume status and chi-square tests to assess for differences in hospital characteristics. The study protocol (#819470) was reviewed and approved by the University of Pennsylvania Institutional Review Board.

## Results

**Fig. 1:**
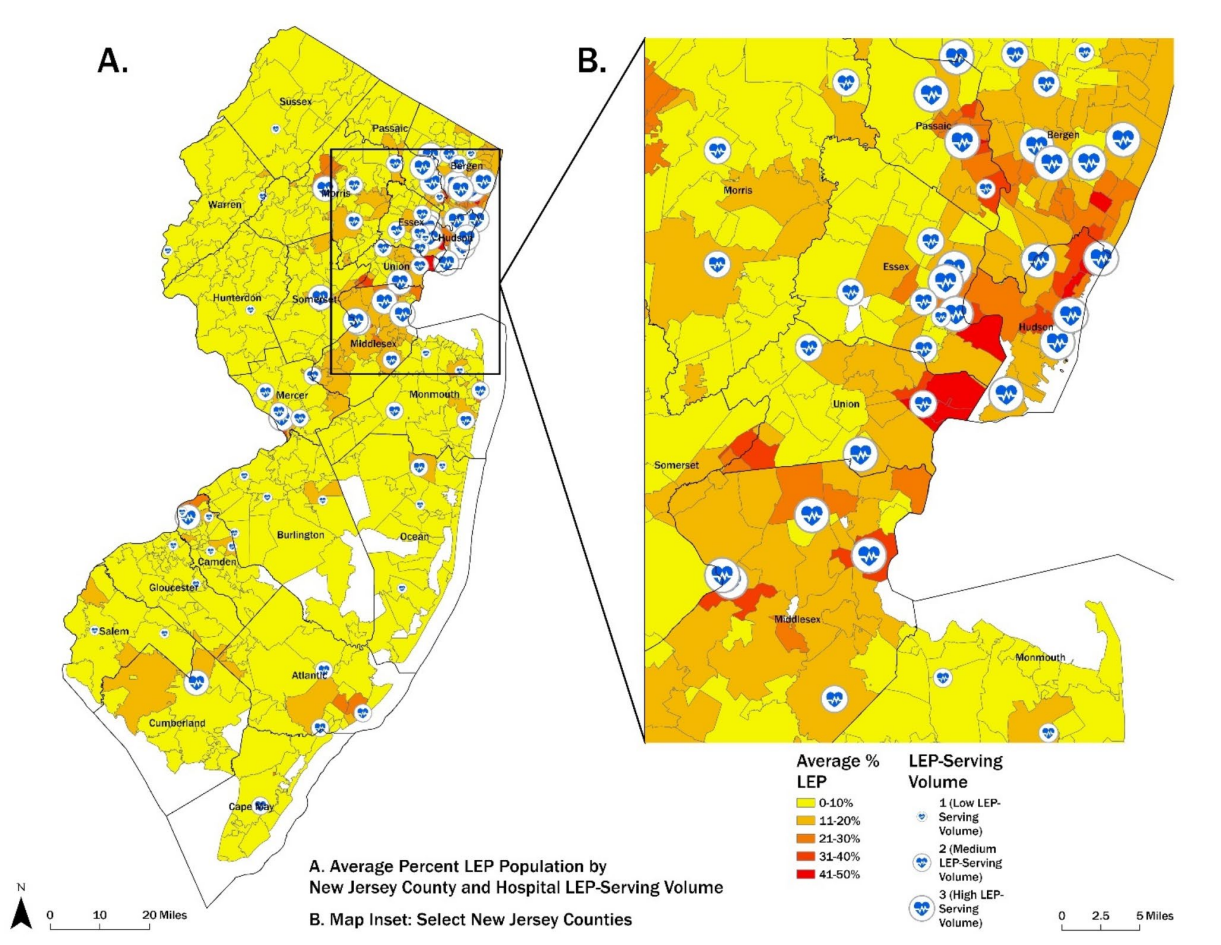
Average percent LEP population by New Jersey county and hospital LEP-Volume. Larger map displays 73 NJ hospitals in our sample. Map inset displays select 37 hospitals across nine counties with high average % LEP. Low LEP volume = 0.02–6.4%, middle LEP volume = 6.6–17.4%, high LEP volume = 17.5–88.1

Most high-LEP volume hospitals are in ZCTAs with higher LEP populations (i.e., the hospital’s patient demographics are reflective of the community in which the hospital is located). However, our results also show instances of segregation in hospital care delivery such that LEP patients forgo care at geographically closer hospitals to instead receive care at farther-distanced, high-LEP volume hospitals (Fig. 1). High-LEP volume hospitals are more likely to be larger, high-technology, teaching hospitals than low-LEP volume hospitals, although these differences were not statistically significant (Table 1). High-LEP volume hospitals on average were lower quality; defined by having higher 30-day hospital-wide readmission rates (20.1%, SD 6.9) compared to middle (15%, SD 6.7) and low-LEP volume hospitals (11.3%, SD 6.8, *p* < .001) (Table 1).

**Table 1:** Hospital characteristics of 73 New Jersey study hospitals by LEP-serving volume

	All hospitals (*n* = 73)	High LEP-serving volume (*n* = 12)	Middle LEP-serving volume (*n* = 24)	Low LEP-serving volume (*n* = 37)	*p*-value
Hospital characteristic					
Average % LEP Served, % (SD) Range of % LEP Served	11.8 (15.9) 0.2–88.1	39.1 (24.1) 17.5–88.1	11 (3) 6.6–17.4	3.4 (1.7) 0.02–6.4	< 0.001
Teaching status^1^, *n* (%)					0.560
No	29 (39.7)	3 (25)	8 (33.3)	18 (48.7)	
Minor	32 (43.8)	6 (50)	12 (50)	14 (37.8)	
Major	12 (16.4)	3 (25)	4 (16.7)	5 (13.5)	
Technology status^2^, *n* (%)					0.800
Not-High	55 (75.3)	9 (75)	17 (70.8)	29 (78.4)	
High	18 (24.7)	3 (25)	7 (29.2)	8 (21.6)	
Bed size, *n* (%)					0.602
<=100 Beds	5 (6.9)	0 (0)	2 (8.3)	3 (8.1)	
101–250 Beds >250 Beds	25 (34.3) 43 (58.9)	4 (33.3) 8 (66.7)	6 (25) 16 (66.7)	15 (40.5) 19 (51.4)	
Quality outcome^3^ Hospital-Wide 30-day Readmission Rate, mean (SD)	14 (7.5)	20.1 (6.9)	15 (6.7)	11.3 (6.8)	< 0.05

*Notes.* LEP = limited English proficiency; SD = standard deviation; Chi-square tests were performed for categorical variables (i.e., teaching status, technology status, bed size) and global analyses of variance (ANOVA) tests were performed for continuous variables (i.e., average % LEP served); ^1^= Non-teaching: have no residents/fellows, Minor teaching: have a ratio of < 1:4 medical residents/fellows per bed, Major teaching: have a ratio of > = 1:4 medical residents/fellows per bed; ^2^= Not High-tech: do not have the capacity to perform open-heart surgery or other major organ transplantation, High-tech: have the capacity to perform open-heart surgery or other major organ transplantation; ^3^=Sample of 62 hospitals due to missing data from CMS Hospital Compare dataset

## Discussion

LEP patients may be forgoing care at geographically closer, higher quality hospitals and pursuing farther-distanced, higher-LEP serving hospitals that appear to have worse hospital readmission quality outcomes. High-LEP volume hospitals had 30-day hospital-wide readmission rates that were nearly twice as high as those of low-LEP volume hospitals. LEP patients, who are also more likely to be uninsured and undocumented [4] may prioritize hospital selection based on availability of language services, bilingual staff, provider referral patterns, experiences of discrimination, or insurance coverage. Previous qualitative studies reinforce this study’s findings by identifying that patients with LEP will go to health care points of care where they feel more likely to encounter a language concordant provider [8]. Although this study is limited to pre-pandemic 2016 data, the age of the data is likely not a limitation since research shows much of the progress made around patient safety and quality of care in the past twenty years has been reversed following the COVID-19 pandemic [9], with Hispanic populations bearing the weight of some of the worst outcomes during this time [10]. Therefore, it is plausible that disparities identified in our study persist today and may even be worse following the effects of the pandemic which left many hospitals and clinicians burnt out and under resourced. To work towards equity in hospital care quality and patient outcomes, future studies should examine which hospital attributes draw LEP patients to seek care in those settings.
